# Editorial: Community series in psychocardiology: exploring the brain-heart interface, volume III

**DOI:** 10.3389/fpsyt.2026.1892267

**Published:** 2026-07-13

**Authors:** Marlies Alvarenga, Kai G. Kahl

**Affiliations:** 1 Institute of Health and Wellbeing, Federation University, Berwick, VIC, Australia; 2 Victorian Heart Institute, Melbourne, VIC, Australia; 3 Medizinische Hochschule Hannover, Hanover, Germany

**Keywords:** adverse childhood experience, blood pressure variability, insomnia, job strain, physical activity, psychocardiology, sleep duration, women heart health

Modern cardiology is defined by precision. It visualises coronary anatomy with remarkable clarity, intervenes on arrhythmogenic substrates with increasing sophistication, and deploys pharmacological strategies with measurable efficacy. Yet this precision remains, in important respects, incomplete. Cardiovascular disease continues to be approached predominantly through a lesion-centred paradigm, one that privileges structural and biochemical abnormalities while under-recognising the biopsychosocial context that shapes disease onset, trajectory, and recovery.

The seven papers in this Part III of The Heart and Mind series converge on a single, clinically consequential proposition: cardiovascular risk and recovery are not solely determined by biological states measured in the present, but are constructed across time through interacting biopsychosocial mechanisms. This is not an argument for diluting cardiology’s technical focus. It is an argument for extending its evidentiary boundaries in ways that improve explanatory power and clinical effectiveness.

A defining strength of this volume lies in how it traces the temporal architecture of cardiovascular risk. Yu et al., drawing on UK Biobank data, move the discussion upstream. Their analysis does not simply show that childhood maltreatment is associated with adult cardiovascular disease; it identifies depressive symptoms as a partial mediator of this relationship. In doing so, Yu et al. shift the clinical narrative. This finding is particularly important because it moves beyond simple association. It begins to show a pathway, where early adversity contributes to affective disturbance, which in turn contributes to cardiovascular burden.

If Yu et al. move the field upstream, Ruan et al. introduce balance into the system. Using NHANES data and the Life’s Essential 8 framework, they demonstrate that better cardiovascular health is associated with lower odds of depressive symptoms, with particularly strong effects in women. Ruan et al. are careful in their interpretation, yet their findings resist a unidirectional model. The relationship between mental and cardiovascular health is reciprocal, dynamic, and mutually reinforcing.

This reciprocity carries practical consequences. Interventions aimed at physical health, physical activity, sleep, metabolic control, may yield psychological benefits, while interventions targeting depression and distress may enhance adherence, engagement, and ultimately cardiovascular outcomes. Ruan et al. do not overstate the case, but they make it difficult to sustain the fiction that these domains can be managed independently.

Where Yu et al. and Ruan et al. establish temporal and reciprocal structure, Stapel et al. introduce a neglected, but clinically tractable, mechanism, sleep. Their review is notable for its restraint. Rather than positioning insomnia as an abstract concern, Stapel et al. document its prevalence in cardiac populations and its association with hospitalisation, functional impairment, and mortality. They also expose a familiar clinical tension. While pharmacological options exist, their safety and comparative effectiveness in cardiac populations remain uncertain.

Stapel et al.‘s contribution is strongest where it is most pragmatic. Their support for cognitive behavioural therapy for insomnia as a first-line treatment is not ideologically driven, but empirically grounded. It offers a low-risk intervention aligned with the needs of cardiac patients. Yet, as they note, implementation is constrained, not by lack of evidence, but by workforce capability and limited integration of sleep science into clinical training. In this respect, Stapel et al. highlight a broader issue, the gap between what is known and what is structurally possible within current models of care.

Their second study (Stapel et al.), in adults with congenital heart disease, sharpens this further. The association between shorter sleep duration and increased epicardial adipose tissue introduces a mechanistic link between behaviour and cardiometabolic risk. Taken together, these papers suggest that sleep should be treated as a modifiable risk factor, not merely a quality-of-life concern.

Lutchman et al. extend the discussion into physiological dynamics. The finding that variability is more strongly associated with anxiety than with depression points to the importance of autonomic regulation as a potential pathway linking psychological states and cardiovascular function. This is a more nuanced view than traditional models that focus on static measurements such as mean blood pressure. It suggests that variability, fluctuation, and regulatory capacity may be as important as absolute levels.

For cardiology, this opens a line of inquiry into how psychological interventions might influence physiological variability. If anxiety is associated with greater blood pressure variability through autonomic mechanisms, then interventions targeting anxiety, whether behavioural or pharmacological, may have measurable cardiovascular effects. This is precisely the kind of integrative hypothesis that psychocardiology is positioned to test.

Where individual-level mechanisms dominate much of the series, Alhajaji et al. shift the frame outward. Their systematic review of healthcare workers situates cardiovascular risk within organisational and societal structures. Long working hours, shift work, and job strain are not incidental stressors, they are patterned exposures with measurable cardiovascular consequences. This is a reminder that cardiovascular disease is not only a matter of individual behaviour or biology, but is also shaped by the conditions under which people live and work.

For the medical profession, this finding carries a particular resonance. Healthcare systems that demand sustained high workloads and irregular hours from their workforce may inadvertently contribute to the very conditions they seek to treat. Addressing cardiovascular risk, therefore, requires not only patient-level interventions, but also organisational and policy-level changes.

Herrmann et al., through the TEACH trial, bring these strands into applied clinical practice. Their stepwise screening model identifies patients with co-occurring psychological distress and poorly controlled cardiovascular risk factors, and in doing so reveals patterns that are both familiar and insufficiently addressed. Women within the cohort were older, more socially isolated, and less physically active, illustrating how psychosocial and behavioural vulnerabilities cluster in ways that amplify risk.

Herrmann et al. resist the temptation to overgeneralise. Instead, they point towards targeted, sex-sensitive interventions that address the conditions shaping behaviour. Their work is instructive precisely because it is operational. It demonstrates how psychosocial and behavioural determinants can be identified, stratified, and integrated into care pathways without disrupting clinical workflow. For women, specifically, this may include strategies to reduce social isolation and increase engagement in physical activity. More broadly, the trial highlights the role of psychological, social, and behavioural determinants in cardiovascular outcomes.

Taken together, these seven papers do not argue for a radical redefinition of cardiology, but for a disciplined expansion of its scope. They suggest that cardiovascular disease is best understood as the product of interacting systems, biological, psychological, behavioural, and social, operating across time. This does not diminish the importance of traditional risk factors, but situates them within a broader framework.

It is at this point that the relevance of the recent 2025 ESC Clinical Consensus Statement becomes clear (1). The document does not introduce a new idea so much as consolidate one, that mental and cardiovascular health exist within a multidirectional system, and that effective care requires their integration. What is striking is not simply the alignment, but the degree of convergence. The themes emerging across the seven papers, early life determinants, bidirectionality, sleep, autonomic regulation, psychosocial stress, and structured screening, map directly onto the domains identified within the ESC framework.

The ESC position is, in this sense, both confirming and clarifying. It formalises the need for systematic screening of mental health conditions, including depression, anxiety, and sleep disturbance, not as an adjunct, but as part of routine cardiovascular care. It articulates the role of multidisciplinary collaboration through the “Psycho-Cardio team”, integrating cardiology, psychology, psychiatry, and allied health into coordinated models of care. And it advances a stepped approach to intervention, in which care is proportionate, targeted, and responsive to clinical need rather than uniformly applied.

Perhaps most importantly, the ESC document identifies what has long been an implicit limitation in cardiovascular care, the absence of structured integration. As outlined in the consensus statement, current models are characterised by insufficient screening, limited awareness of mental health burden, and fragmentation between disciplines. The seven papers in this series do not merely describe these gaps, they provide empirical pathways through which they might be addressed.

There is, however, a discipline required in how this integration is pursued. The argument is not for indiscriminate expansion. Not every patient requires intensive psychological intervention, and not every psychosocial variable carries equivalent clinical weight. The task is to identify where psychological and behavioural factors materially influence risk, adherence, and recovery, and to intervene with precision.

In this respect, the future of cardiovascular care is not broader, but sharper. It requires a shift in clinical reasoning, away from categorical distinctions between “cardiac” and “non-cardiac”, towards a process-oriented understanding of risk and recovery. The relevant question is no longer whether a factor belongs within cardiology, but whether it contributes to the mechanisms that generate disease.

The seven papers in this series, taken together, offer a compelling answer. They show that psychological, behavioural, and social processes are not peripheral to cardiovascular disease, they are constitutive of it. Indeed, cardiovascular disease does not occur in isolation from the conditions of a person’s life, and its treatment should not proceed as though it does. The opportunity before the field is not to dilute its technical sophistication, but to extend it, to align its precision with the full complexity of the systems it seeks to treat.
